# What spatial omics is teaching us about field cancerisation in prostate and bladder cancer

**DOI:** 10.1111/bju.16830

**Published:** 2025-06-25

**Authors:** Matthew H. V. Byrne, Thineskrishna Anbarasan, Lisa Browning, Dan J. Woodcock

**Affiliations:** ^1^ Nuffield Department of Surgical Sciences University of Oxford Oxford UK; ^2^ Department of Cellular Pathology Oxford University Hospitals NHS Foundation Trust Oxford UK

**Keywords:** spatial transcriptomics, spatial proteomics, spatial genomics, spatial metabolomics, field cancerisation, field change, field effect, field defect, bladder cancer, prostate cancer

## Abstract

**Background and Objectives:**

Field cancerisation is the process that results in a group of cells acquiring some of the phenotypic changes of cancer prior to transformation into cancer. Clinically, an important challenge remains the ability to distinguish clonal lineages and microenvironments within cancerised fields that will remain indolent from those that will progress to malignant transformation. Spatial ‘omics’ can help us investigate genetic, epigenetic, transcriptomic, proteomic, and cellular microenvironments that transform normal cells into a cancerised field, and subsequently into cancer. In this review, we will discuss how spatial omics techniques have expanded our understanding of field cancerisation in prostate and bladder cancer, and the challenges associated with this research.

**Methods:**

We identified key articles relating to field cancerisation in bladder and prostate cancer. Special emphasis was placed on studies that used modern spatial profiling technologies and studies that were designed to investigate changes within normal tissue rather than simply using it as a control for tumour tissue.

**Results:**

Spatial omics research into field cancerisation has identified interesting early findings that have informed our understanding of: transformation of the benign epithelium and mechanisms of intra‐prostatic clonal expansion for prostate cancer; clonal expansion within the normal urothelium; mutations that are unique to cancerised fields within the bladder; and how field cancerisation may prime the urothelium for cancer transformation.

**Conclusions:**

Spatial omics profiling of field cancerisation can inform risk stratification and personalised treatment options. However, there are a number of challenges associated with the technologies that must be overcome before the potential of spatial omics can be fully realised in clinical practice.

**Abbreviations**
ATAC‐seq: assay for transposase‐accessible chromatin with sequencing
CNV: copy number variation

## Introduction

**Fig. 1:**
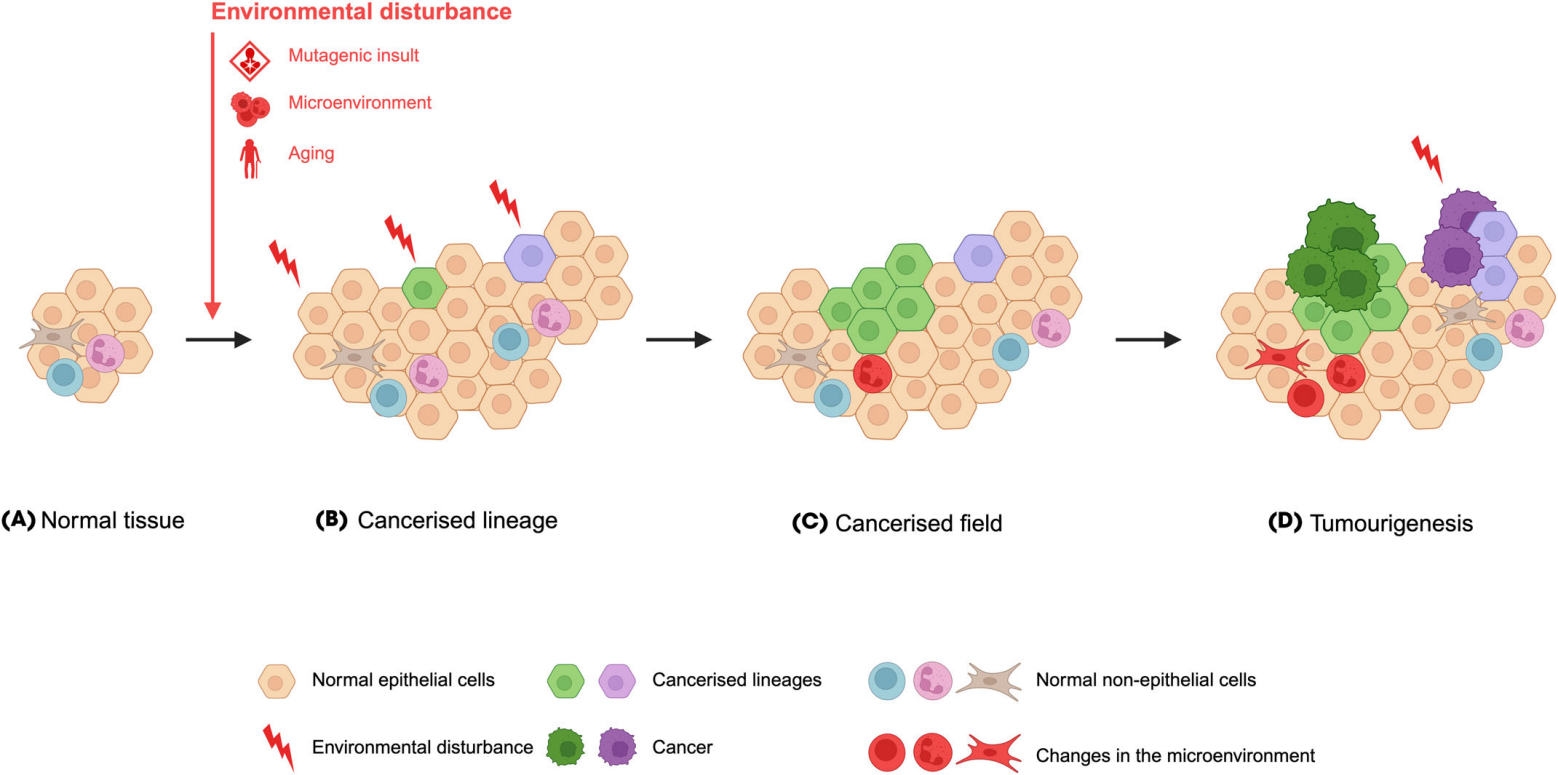
The process of field cancerisation. (**A**) Normal tissue is exposed to an environmental disturbance such as a mutagenic insult, a change in the cellular microenvironment, or accrual of somatic mutations due to aging. (**B**) This causes some cells to acquire some of the phenotypic changes of cancer. (**C**) These changes may provide a growth or survival advantage, which allows some cancerised lineages to expand into a cancerised field. The cancerised field may subsequently influence its microenvironment, or cancerised cells may develop intrinsic changes that move their evolution towards cancer. (**D**) A cell within the cancerised field acquires additional changes which permit transformation into cancer; this can influence the cellular microenvironment further. Some cell lineages may require further exposure to environmental disturbances and polyclonal cancer formation can occur. Created using BioRender.

Field cancerisation (also known as field effect, field change, and field defect) refers to the process that results in a group of cells acquiring some of the phenotypic features of cancer, prior to transforming into cancer [1]. Mutagenic insults, microenvironmental changes, and age‐related changes may cause field cancerisation [1]. These environmental disturbances induce changes in normal cells, including genomic changes, transient changes such as epigenetic changes [2], and context‐specific changes, such as a specific cellular microenvironment [3]. These cancerised cells include histologically normal cells as well as cells with morphological changes such as dysplasia, hyperplasia, or metaplasia [1]. Cancerised cells can clonally expand into a cancerised field through displacement of normal epithelial cells, for example, a monoclonal field may expand after a cancer stem cell has acquired changes that provide a growth or survival advantage [4]. Some diseases have many small clonal populations [5], whereas others demonstrate domination by a limited number of clones [6]. Alternatively, a polyclonal field may develop through multiple exposures to a mutagen and different clones evolving separately over time [1]. Many tissues can be affected by field cancerisation including skin, lung, oesophagus, colon, prostate, and bladder, and the size of the cancerised field can vary by tissue type – from a small number of cells to the whole organ [7, 8, 9]. Field cancerisation, therefore, can be viewed as a dynamic process that results in areas of tissue of varying size, that can include mono‐ or polyclonal cell lineages on an evolutionary trajectory towards malignancy (Fig. 1; Box 1).

Box 1 **Key definitions.**

**Field cancerisation:** the process that transforms a normal cell into a cancerised field.

**Cancerised lineage:** a clonal population that has acquired some of the phenotypic changes of cancer. These cells have not yet transformed into cancer.

**Cancerised field:** the area of tissue consisting of a cancerised lineage.

Clinically, an important challenge remains distinguishing clonal lineages and microenvironments within cancerised fields that will remain indolent from those that will progress to malignant transformation. This is particularly important in bladder and prostate cancer where there is a high cost associated with surveillance, and a risk of under‐ or overtreatment, respectively. Risk and treatment stratification remains a challenge [10] and is compounded by extensive molecular heterogeneity, combined with both diseases’ multifocal phenotype, which renders precision diagnosis and treatment more challenging [11, 12].

Modern spatial ‘omics’ methods can help us investigate genetic, epigenetic, transcriptomic, proteomic, and cellular microenvironments that transform normal cells into a cancerised field, and subsequently into cancer. In this review, we will discuss how these techniques have expanded our understanding of field cancerisation in prostate and bladder cancer, potential future directions, and the challenges associated with this research (Box 2).

Box 2 **Key references for field cancerisation in bladder and prostate cancer.**

Erickson A, He M, Berglund E et al. Spatially resolved clonal copy number alterations in benign and malignant tissue. *Nature* 2022; 608: 360–7 [13]

Zhang N, Harbers L, Simonetti M et al. High clonal diversity and spatial genetic admixture in early prostate cancer and surrounding normal tissue. *Nat Commun* 2024; 15: 3475 [14]

Li R, Du Y, Chen Z et al. Macroscopic somatic clonal expansion in morphologically normal human urothelium. *Science* 2020; 370: 82–9 [15]

Lawson ARJ, Abascal F, Coorens THH et al. Extensive heterogeneity in somatic mutation and selection in the human bladder. *Science* 2020; 370: 75–82 [16]

Majewski T, Yao H, Bondaruk J et al. Whole‐organ genomic characterisation of mucosal field effects initiating bladder carcinogenesis. *Cell Rep* 2019; 26: 2241–56.e4 [17]

## Spatial Omics Techniques

**Fig. 2:**
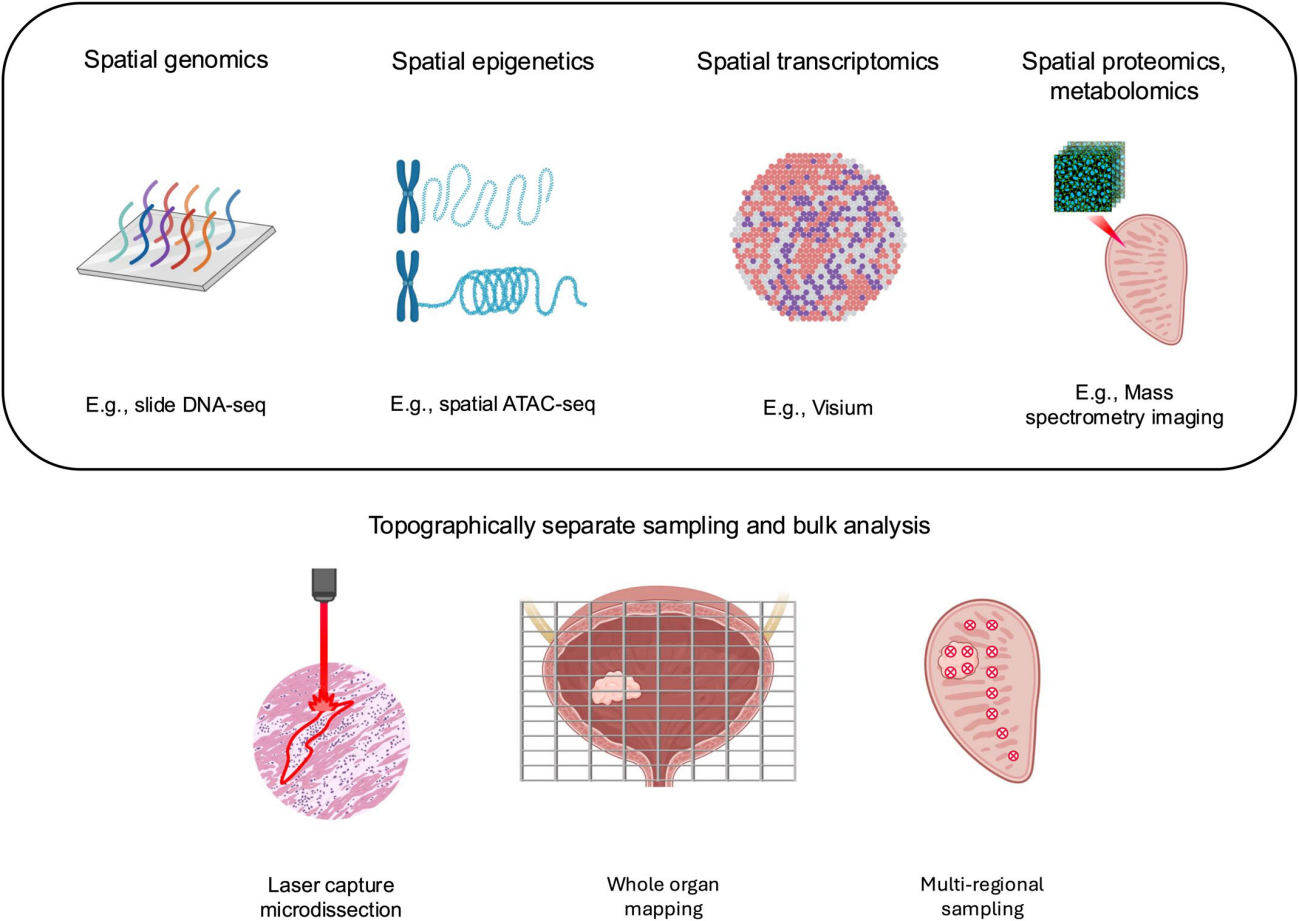
Spatial omic technologies which could be applied to investigate field cancerisation in prostate and bladder cancer. The top box displays examples of spatial techniques. The bottom section displays topographically separate sampling through laser capture microdissection, whole organ mapping, or multi‐regional sampling, followed by bulk analysis of these samples. Figure created using BioRender. ATAC‐seq, assay for transposase‐accessible chromatin with sequencing; DNA‐seq, DNA sequencing.

A range of technologies now enable profiling of protein expression, transcriptome, epigenome, metabolomics and DNA with respect to their spatial co‐ordinates (Fig. 2). These technologies are suited for a spectrum of applications guided by input tissue type, resolution of genomic profiling, throughput and cost. Spatial proteomics and spatial transcriptomics are currently the most common and well‐developed spatial techniques used. We have provided a summary of their advantages and limitations in Table 1.

**Table 1:** Advantages and limitations of leading spatial proteomic and spatial transcriptomic technologies.

Technology	Method	Tissue	Advantages	Limitations	References
Spatial proteomics					
PhenoCycler Fusion (CODEX ‐ Co‐Detection‐by‐InDEXing)	Antibody tagged with DNA barcodes to detect target proteins (up to hundreds)	FFPE, FF	Highly multiplexed DNA barcode allows precise targeting Single‐cell resolution	High cost Dependence on antibody	[18]
Immunohistochemistry	Antibody linked with enzyme or dye to detect target proteins	FFPE, FF	Low cost Widely available	Limited multiplex Subjective interpretation	[19]
Laser capture microdissection coupled mass spectrometry	Laser capture microdissection used to isolate regions of interest which are analysed with mass spectrometry to quantify protein abundance	FFPE, FF	Precise quantification High specificity Multiple targets can be studied, suitable for biomarker screening	High cost Spatial resolution limited by microdissection area	[20]
Imaging mass cytometry	Metal‐tagged antibodies to targets (~40) which are released on laser ablation and analysed by mass spectrometry	FFPE, FF	Single‐cell resolution Highly multiplexed No spectral overlap	High cost Complex equipment Limited tissue size	[21]
Digital spatial profiling	Fluorescence microscopy marked regions of interest exposed to DNA barcode targeting probes tagged with photocleavable linkers. Ultraviolet light exposure releases the tags allowing sequencing‐based quantification.	FFPE, FF	Highly multiplexed Can also detect RNA Single‐cell resolution	High cost Dependence on antibody	[22]
Multiplexed ion beam imaging	Mass spectrometry used to measure targets (~50 s) linked with antibody tagged with elemental mass	FFPE	No spectral overlap Precise quantification High resolution	High cost Technically complex	[23]
Spatial transcriptomics					
Visium and Visium HD	Slides with spatially barcoded spots (55 μm [Visium] or 2 μm [Visium HD]) which capture RNA for NGS	FFPE, FF	Whole transcriptome Numerous analysis tools for Visium Unbiased	High cost Resolution dependent on spot size	[24, 25]
MERSCOPE	Based on MERFISH (Multiplexed error‐robust fluorescence *in situ* hybridisation). *In situ* hybridisation technique where fluorophore signal from tagged RNA is detected	FFPE, FF	High spatial resolution Highly multiplexed	Targets about 1000 genes High cost Sensitivity dependent on probe design Low throughput	[26]
Stereo‐Seq (Spatial transcriptomics by sequencing)	Spatially barcoded DNA nanoballs are used to capture RNA, which are then sequenced	FF	Whole transcriptome Large detection area High resolution	High cost Low capture efficiency	[27]
Slide‐seqV2	Spatially barcoded beads used to capture RNA which are sequenced	FF	Whole transcriptome High resolution High throughput	High cost Low capture efficiency	[28]
Xenium	Probes are hybridised to target RNA which are amplified. These are tagged with fluorescent probes which are imaged	FFPE, FF	High resolution Speed of data generation	Targeted gene panel High cost	[29]
STARmap	Probes bind to mRNA and these are amplified *in situ*, stabilised within a hydrogel which enables three‐dimensional imaging of these amplified spots	FF	Three‐dimensional (150‐μm thickness) High resolution	Targeted gene panel Technically complex	[30]
Sequential fluorescence *in situ* hybridisation (seqFISH+)	Fluorescent probes sequentially bind to target RNA, multiple probes used for each RNA to act as a temporal barcode	FF	10 000 gene coverage High resolution	Requires multiple hybridisation rounds Low throughput	[31]
DBiT‐seq and Pathology‐Compatible DBiT‐seq (Deterministic Barcoding in Tissue for Spatial Omics Sequencing)	Barcodes applied via a 100 × 100 channel microfluidic device with a spot size of 20–50 μm	FF, FFPE	Whole transcriptome Unbiased Can include a protein panel (DBiT‐seq)	Resolution dependent on spot size	[32, 33]

FF, fresh frozen; FFPE, formalin‐fixed paraffin‐embedded; NGS, next‐generation sequencing.

### Spatial Proteomics

Spatial proteomics enables understanding of protein distribution, interaction and dynamics at a subcellular level, which is important for interrogating biological pathways involving protein localisation implicated in carcinogenesis and its prognosis. In general, spatial proteomics relies on three complementary approaches: (1) mass spectrometry analysis to quantify fractionated organelles from subcellular compartments to generate an organellar map using bioinformatic analysis; (2) labelling proteins based on proximity to analyse protein interaction networks; and (3) *in situ* visualisation of protein localisation using affinity reagents such as fluorescent markers and antibodies [34].

### Spatial Transcriptomics

Spatial transcriptomics allows characterisation of messenger RNA (mRNA) expression with reference to spatial loci within the tissue and is generally classified into imaging‐based and sequencing‐based methods.

Imaging‐based methods profile the transcriptome by first imaging mRNAs *in situ* using microscopy. Two approaches can then be used to discern between mRNA species: *in situ* hybridisation and *in situ* sequencing. *In situ* hybridisation utilises hybridisation of single‐stranded mRNAs to complementary single‐stranded probes containing a chromogenic group [35]. The *in situ* sequencing approach involves the use of probes to profile short segments of amplified transcripts linking them to different fluorophores, allowing transcript identification [36].

Sequencing‐based methods involve mRNA extraction for sequencing, with preservation of their spatial information mainly via direct recording of loci using microdissection or microfluidics [37, 38], or ligation of mRNA to spatially barcoded probes within a microarray [25]. Spatial transcriptomic technologies allow study of the spatial associations of cells or regions of interest with transcriptomic changes. By considering the physical distance between cells and leveraging a range of computational solutions, cell–cell interactions can be uncovered. Spatial transcriptomics data can be represented in two‐dimensional space, which can be analysed in conjunction with serial sections to identify landmarks as part of a common coordinate framework to perform three‐dimensional reconstruction. Together, spatial transcriptomics provides multi‐dimensional insights into the spatially resolved biological landscape of the tissue of interest and is an essential tool to understand field effects in cancer.

### Spatial Epigenetics

Epigenetic changes are generally divided into DNA methylation and histone tail modifications that affect chromatin dynamics and gene expression. Spatial epigenomics is an emerging technology in comparison to spatial proteomics and spatial transcriptomics, with comparatively few studies using the technology. One approach is to perform simultaneous spatial assay for transposase‐accessible chromatin with sequencing (ATAC‐seq) and RNA sequencing, whereby profiling of chromatin assembly and mRNA expression is performed in a spatially resolved manner [39]. Using co‐expressed spatial barcodes and layering the epigenome and transcriptome, this technology allows interrogation of accessible chromatic peaks, establishing correlations with transcriptional regulation at close to single‐cell level [40].

### Spatial Genomics

Slide‐DNA‐seq is a novel approach for spatially resolved DNA sequencing. It has been developed using barcoded technology to facilitate quantification of mutant allele frequencies alongside their spatial context [41]. This addresses the limitations of approaches such as laser capture microdissection, which requires manual selection of cells and is thus more suitable for late‐stage cancers [41]. However, slide‐DNA‐seq requires validation and likely has similar limitations to Slide‐seq, which is used for RNA sequencing, including high cost and low capture efficiency.

### Spatial Metabolomics

Metabolites and lipids are gaining increasing attention for their role in fuelling tumourigenesis. Spatial metabolomics is a novel approach which uses imaging mass spectrometry to identify metabolites within the tissue architecture and has exciting applications, such as detecting the localisation of drug metabolites [42]. However, it is still within the early phase of adoption as a technology, and can be limited by the structure of tissues (e.g., fat content, porosity, and tissue density), and requires fresh‐frozen tissue due to enzyme degradation of metabolites [42].

### Topographically Separate Sampling and Bulk Analysis

The advent of many of these technologies is recent. Bulk omic profiling of multi‐regional sampling has previously been used: representative samples are taken from various spatially distinct regions in the tissue, and bulk omic profiling is performed. The main techniques used for topographically separate sampling and bulk analysis are laser capture microdissection, whole organ mapping, and multi‐regional sampling. Laser capture microdissection uses a laser to remove user‐selected regions of interest from a tissue slide; these samples can then be sent for bulk analysis. For example, a small number of epithelial cells can be isolated for further analysis including bulk DNA and RNA sequencing, and methylation analysis [16]. Whole organ mapping uses a grid of 2 × 1‐cm wells to divide the whole organ, and RNA, DNA, proteins, or methylation changes can be isolated within each well [43]. This can then be combined with histological analysis of each section to annotate the areas as normal, low or high grade (including severe dysplasia and carcinoma *in situ*), or cancer [43]. Multi‐regional sampling involves sampling regions of interest from tissue (e.g., with a punch biopsy or scalpel) and then performing bulk analyses on these samples.

Table 2 shows the spatial omic technologies that have been used to investigate field cancerisation in prostate and bladder cancer and the key findings from these studies. In the following sections we discuss the biological findings in detail.

**Table 2:** Key studies investigating field cancerisation in prostate and bladder cancer according to the spatial omic technology used in each study.

Study	Spatial omic method	Key findings
Prostate		
Cooper et al. (2015) [44]	Topographically separate sampling	Whole genome sequencing of separate regions within axial prostate sections revealed mutations in histologically benign areas distant from the cancer foci
Yang et al. (2013) [45]	Topographically separate sampling	Epigenetic changes were compared between tumour‐associated and non‐tumour‐associated tissue, which was histologically benign. Tumour‐associated tissue that was adjacent (2 mm) and distant (>1 cm) from tumour foci had similar DNA methylation changes
Rao et al. (2024) [46]	Topographically separate sampling	Whole genome sequencing of topographically separate areas revealed specific clones within the prostate, which metastasised to lymph nodes. Evidence of intra‐prostatic spread of tumour whilst accumulating genomic changes were observed
Zhang et al. (2024) [14]	Topographically separate sampling	Prostate midsection divided into ~ 125 mm^3^ regions used for bulk sequencing. Somatic copy number changes were observed to be widespread in tumour and normal areas across the prostate with localised tumour
Hirz et al. (2023) [47]	Topographically separate sampling, spatial transcriptomics (Slide‐seqV2)	Spatial transcriptomic analyses combined with single‐cell RNA sequencing revealed an immunosuppressive environment (exhausted T cells and suppressed myeloid population) associated with localised prostate cancer. The prostate tumour microenvironment exhibited high angiogenic gene expression
Erickson et al. (2023) [13]	Spatial transcriptomics (Visium)	Inferred copy number changes from axial prostate wide spatial transcriptomics revealed mutations in histologically benign regions which shared phylogenetic relationship to tumour foci
Bladder		
Gaisa et al. (2011) [48]	Laser capture microdissection	Clonal patches exist in the bladder urothelium ranging in size from 30 μm to 4.7 mm, likely supported by a single stem cell
Li et al. (2020) [15]	Laser capture microdissection	Clonal patches can be up to 2 cm in size. Independent clones may be found in the same patient
Strandgaard et al. (2020) [49]	Laser capture microdissection	Shared and unique mutations can be found in both tumour and normal samples
Lawson et al. (2020) [16]	Laser capture microdissection	Positive selection of genes can occur in normal tissue Only a small number of bladder cancer driver gene mutations separate carcinoma *in situ* and normal samples
Majewski et al. (2008) [50]	Whole organ mapping	Single RB1 allele loss can be present across large areas of the normal urothelium. Loss of both RB1 alleles was associated with carcinoma *in situ* and bladder cancer
Bondaruk et al. (2022) [43]	Whole organ mapping	Mutations in bladder cancer can be divided into: Mutations unique to single samples β mutations which occur in a ‘dormant phase’, where mutations slowly accrue over 10–15 years α mutations which occur in a ‘progressive phase’, where mutations occur at a high frequency over 1–2 years

## Methods

We performed a systematic search of Medline, using the criteria shown in Appendix S1, to identify articles relevant to field cancerisation in bladder and prostate cancer. We reviewed human studies that were available in English, and we excluded review articles. We did not use a publication year limit, but most studies prior to 2010 were not relevant due to the nature of this review. As this is a narrative review, we did not include all articles reviewed, rather we included key articles, and special emphasis was placed on studies that used modern spatial profiling technologies and studies that were designed to investigate changes within normal tissue rather than simply using it as a control for tumour tissue.

## Spatial Profiling of Field Cancerisation in Prostate Cancer

### Transformation of Benign Prostate Epithelium

A prerequisite for field cancerisation is for a cell population to undergo a transformative event attaining some, but not all, of the phenotypic features of malignancy. Genomic changes have been observed in the prostate epithelium in the absence of any morphological transformation. Through massively parallel whole genome sequencing of regionally sampled prostate cancer and morphologically normal tissue, Cooper et al. [44] showed clonal expansions and changes that were consistent with field cancerisation within histologically normal prostate. Epigenetic changes have also been observed in normal prostatic tissue. Through a DNA methylation microarray assay of tumour, normal tissue adjacent to (2 mm) and distant from (10 mm) tumour foci, Yang et al. [45] demonstrated widespread epigenetic defects with similar burden of methylation events across different tissue locations. DNA hypomethylation was also observed in prostatic intraepithelial neoplasia (a pre‐malignant lesion with atypical cytomorphology that is confined to the prostatic ducts and acini) [45]. Leveraging spatial transcriptomics, Erickson et al. [13] demonstrated intra‐prostatic tumour heterogeneity, with evidence of clonal expansion driven through acquisition of copy number alterations, and with some copy number changes shared with histologically benign regions. Thus, histologically benign areas of prostate tissue may display both genetic and epigenetic changes consistent with field cancerisation, which may subsequently be a precursor to the development of prostate cancer.

Erickson et al. [13] also demonstrated that the clonal populations within histologically benign areas were enriched for genes associated with oxidative phosphorylation, mitochondrial energy metabolism and protein stabilisation when compared with malignant areas, suggesting a response mechanism to intrinsic and extrinsic cellular stress. Interactions between the tumour microenvironment and the neighbouring cell populations have been shown to promote the transformation to malignancy. Using a combination of single‐cell and spatial transcriptomic sequencing in several cancer samples including prostate cancer, Lior et al. [51] show a spatial gradient in the degree of stress response with proximity to tumour. Hirz et al. [47] characterised prostate cancer and adjacent normal tissue using single‐cell and spatial transcriptomics and demonstrated that prostate cancer foci established an immunosuppressive microenvironment with a high degree of angiogenic gene expression that promotes tumourigenesis and progression amongst the neighbouring cells [47]. Hirz et al. also demonstrated that, within normal tissue adjacent to the tumour, there was loss of glandular architecture and expansion of luminal epithelial cell populations when compared to healthy controls. Additionally, a myeloid‐derived suppressor cell gene signature was significantly higher in tumour, and tumour‐adjacent normal tissue compared with healthy controls [47]. This suggests that cancerised lineages may be able to exert influence on neighbouring cells, creating a pro‐tumorigenic field with an altered immune cell expression that is susceptible to polyclonal expansion with or without morphological changes.

While a cancerised field may include cells with morphological changes such as hyperplasia [1], Chae et al. [52] demonstrated that BPH was not a form of field cancerisation. They profiled somatic mutations in normal prostate and BPH to assess their link to prostate cancer using three‐dimensional mapping and whole genome sequencing. Chae et al. [52] found driver mutations and copy number alterations in both normal and BPH tissues, although these were rare and seldom overlapped with prostate cancer. Interestingly, oncogenic RTK/RAS somatic mutations were observed in one normal prostate sample, suggesting convergence in clonal maintenance. BPH samples had a marginally higher mutation burden, shorter telomeres, and larger clone sizes compared to normal tissue. The BPH genome was overall similar to normal prostate and harboured significant differences from prostate cancer. Thus, while there may be morphological differences between BPH and normal tissue, it is unlikely that BPH is related to tumorigenic processes [52].

### Mechanisms of Intra‐prostatic Clonal Expansion Remain Unclear

Phylogenetic analyses using multi‐regional deep genomic sequencing of archived tissue, combined with histopathological analysis, have been used to explore the evolutionary dynamics of prostate cancer within the prostate. Rao et al. [46] showed intra‐prostatic evolution of tumour clones with distinct morphological features associated with separate evolutionary branches. In one patient, the authors mapped the spread of cancer from the apex of the prostate to the seminal vesicles and characterised genomic changes associated with the adenocarcinoma to amphicrine morphology transformation [46]. This suggests that prostate cancer foci may spread or seed within the prostate through mechanisms which remain unclear, with intraductal spread being proposed as a possibility [46]. Therefore, the multiple polyclonal foci observed in prostate cancer may not be solely explained by field cancerisation.

By dividing the mid‐gland section of a formalin‐fixed paraffin‐embedded prostatectomy specimen into ~125 mm^3^ and performing single‐cell ‘CUTseq’, a novel approach used to detect somatic copy number alterations, Zhang et al. [14] observed the widespread presence of pseudo‐diploid (typically consisting of [sub‐]chromosomal arm deletions) and aneuploid cells (multiple whole chromosome amplifications and deletions) across the specimen, including benign areas distant from tumour. This observation provides support for widespread genomic instability or field cancerisation before cancer development.

In studies of metastatic prostate cancer, only a few new driver events occurred following metastatic dissemination, with the majority of sub‐clonal expansions taking place within the prostate [53]. After metastasis there appears to be relative homogeneity in the genomic profile of lymph nodes with a clonal origin that can be traced back to the prostate [54].

Taking the evidence together, the exact mechanism driving multi‐focality in prostate cancer remains unclear. It could be speculated that field cancerisation results in widespread genomic instability, resulting in the development of cancer clones that can subsequently seed or spread to other regions through the acquisition of a few key driver mutations. A combination of high‐resolution spatial genomic profiling with a temporal component, either including profiling a systematic biopsy specimen with known loci or pseudotime analysis with bioinformatics approaches, such as SpaceFlow, may be valuable to decipher the aetiology of multi‐focality in prostate cancer [55]. Spatial profiling of temporal biopsies, for example, in men on active surveillance who progress to clinically important disease, with the subsequent prostatectomy specimen, can be valuable. This will provide a patient‐level evolutionary trajectory to aid understanding of the influence of accumulating stochastic genomic alterations and/or expansion of a high‐risk clone towards field cancerisation.

## Spatial Profiling of Field Cancerisation in Bladder Cancer

For bladder cancer, the two main spatial techniques that have been used to investigate field cancerisation are whole organ mapping, and laser capture microscopy (Table 2).

### Clonal Expansion within the Normal Urothelium

Spatial techniques have influenced our understanding of clonal expansion of histological normal urothelial cells. Gaisa et al. [48] stained serial histology sections for cytochrome c oxidase (a mitochondria encoded enzyme whose mutations are often age‐related and are not thought to be positively selected for) in 16 cystectomy specimens. Laser capture microdissection was then performed on areas that were positively and negatively stained and the mitochondrial genome was sequenced. This confirmed that in separate patches there were identical mutations within that patch. These clonal units ranged in size from 30 μm (2–3 cells) to 4.7 mm in size. The samples were stained for additional cell type markers, which demonstrated that cells at different stages of urothelial differentiation were present. From this analysis, they demonstrated that the normal mucosa surface comprises patches that are likely supported by a single stem cell and, as there is variability in the sizes of these patches, this suggests that there is expansion or displacement of adjacent stem cells [48].

Exposure to a mutagen (in this case, aristolochic acid) can influence how the cancerised field expands. For one patient who had muti‐regional sampling, the size of the clonally expanded area was at least 2 cm, whereas another patient demonstrated multiple normal and tumour clones. This suggests that clones can evolve independently after the same mutagen exposure [15].

### Mutations Can be Unique to Field Cancerised Areas

Strandgaard et al. [49] identified 13 shared mutations between tumour and normal samples, and 13 mutations that occurred in normal tissue but not found in tumour tissue. There were also different nucleotide changes in tumour and normal tissue, with C>T higher in tumour and C>G higher in normal tissue. To demonstrate this, the authors performed laser capture microdissection, whole exome sequencing, and evaluation of a deep‐target amplicon panel (509 driver genes) of 17 tumour and 27 normal urothelium samples from four patients who underwent radical cystectomy [49]. Another study showed that the number of mutations present in normal tissue may be higher in patients with multifocal disease [56].

In a larger study of 20 patients (five patients with cystectomy for bladder cancer, and 15 deceased transplant organ donors), Lawson et al. [16] used laser capture microdissection targeted sequencing of 321 cancer‐associated genes, and whole exome and genome sequencing. They identified 17 genes that were positively selected for in the normal epithelium. This suggests that these genes provide a competitive advantage for normal urothelial cells. Five of these genes were chromatin remodelling genes, however, many common bladder cancer genes were entirely absent from the samples (e.g., *TP53, FGFR3*). The findings from a comparable study by Li et al. were similar [15]. This implies that these do not confer an advantage to normal urothelium, even though they are important driver mutations in bladder cancer.

### Field Cancerisation May Prime the Urothelium for Cancer Transformation

In the study by Lawson et al. [16], three of the five patients with bladder cancer had concurrent carcinoma *in situ*. The mutational burden of carcinoma *in situ* was slightly higher than normal tissue, and most mutations were shared with the normal distant bladder biopsy except for a few additional bladder cancer driver mutations [16]. This suggests that only a small number of additional steps are required to transform field cancerised areas into bladder cancer.

Indeed, Majewski et al. [50] performed whole organ histological and genetic mapping of five cystectomy samples and single nucleotide polymorphism analysis of RB1 on chromosome 13q14. Loss of one RB1 allele was common across large areas of urothelium in four of the five patients, suggesting clonal expansion, and subsequent loss of the second RB1 allele was common in severe dysplasia, carcinoma *in situ*, or urothelial cancer [50].

Bondaruk et al. [43] performed whole organ mapping to analyse the molecular evolution of bladder cancer due to field cancerisation throughout the bladder in two cystectomy samples. The analysis included bulk RNA sequencing, whole exome sequencing, methylation analysis, and single nucleotide polymorphism based copy number variation (CNV) analysis. Three types of mutations were identified: (1) heterogenous mutations that were limited to single samples, thus, likely to represent individual uroprogenitor cell mutations; (2) α mutations, which occurred at a low frequency over 10–15 years, during a ‘dormant’ phase; (3) β mutations, which occurred at a high frequency over 1–2 years, during a ‘progressive’ phase when bladder cancer likely forms.

The mechanism underlying the transformation of a cancerised field into cancer is not well understood. For example, Li et al. [15] found that copy number changes in normal samples were rare, and suggested these were late changes. Whereas Majewski et al. [17] suggested that, while a few CNV changes were identified in normal tissue, these can be present across large areas of the mucosa and so can be an early change. Bondaruk et al. [43] demonstrated that there were widespread CNV changes in the tumour and surrounding tissue in a clearly demarcated area, but that CNV changes were less common in normal urothelium.

Whether there is a single initiating mutation that drives field cancerisation is also not clear. In two patients, one with luminal urothelial carcinoma and the other with basal urothelial carcinoma, Bondaruk et al. [43] suggested that BAP1 was the founding mutation in luminal disease, and CARPIN1 was the founder mutation in basal disease. Majewski et al. [17] suggested that ACIN1 was a founder mutation, and that KRAS was a key driver for disease progression to severe dysplasia, carcinoma *in situ*, and urothelial cancer.

Epigenetic changes may also have a role in early field cancerisation. Majewski et al. [17] demonstrated widespread methylation changes in the whole organ mapping of one cystectomy sample.

Taking the evidence together, it appears as though field cancerisation does prime the urothelium for transformation into cancer. Although the exact mechanisms underlying this are not fully clear, and differences among studies may represent disease heterogeneity or may be due to the limited number of patients involved.

## Future Directions

There is a wide range of additional spatial omic techniques that could be used to investigate field cancerisation within bladder and prostate cancer. For example, infra‐red matrix‐assisted laser desorption electrospray ionisation mass spectrometry imaging has been used to identify different metabolomic profiles in separate regions of bladder tumours [57]. Spatial techniques could be used to explore similar heterogeneity within cancerised fields. Imaging mass cytometry has been used to identify cancer stem‐like cells, which were located in a stroma‐rich area at the invasive edge of bladder tumours, and were associated with worse prognosis [58]. Spatial techniques could be used to identify high‐risk cancerised lineages. Multi‐regional and longitudinal sampling of cancerised fields could track how both monoclonal and polyclonal cancerised lineages evolve, and the evolutionary steps which transform a cancerised lineage into cancer. However, this may be limited by technical challenges – such as sampling the same location at multiple timepoints; a small cancerised field theoretically could be eliminated through the sampling process.

Many studies of prostate and bladder cancer that do not focus on field cancerisation use adjacent normal tissue as a control for their spatial profiling of tumour tissue; however, there may be shared changes between normal urothelium sampled adjacent to the tumour and tumour tissue that are missed by using this approach [13, 49]. If this is not taken into consideration, the findings of these studies may be limited.

Spatial techniques can be used to define different spatial tumour microenvironment phenotypes, such as: ‘immune excluded’, ‘infiltrated’ and ‘deserted’ areas within bladder tumours [58], and changes in the tumour microenvironment in prostate cancer, such as changes in immune and non‐immune stromal cell populations [59]. A detailed understanding of the cellular environments within a cancerised field could be developed through spatial techniques.

Understanding of the molecular and cellular microenvironment could then be used to understand the biology of field cancerisation and response to treatment. For example, GeoMx digital spatial profiling was used to identify inflamed tumour areas and infiltrated stroma, which was associated with a better response to pembrolizumab in BCG immunotherapy‐unresponsive tumours [60]. Within prostate cancer, longitudinal sampling of treatment‐resistant disease combined with spatial transcriptomics and immunohistochemistry has been used to help understand mechanisms for treatment resistance, such as the location of active androgen receptors relative to the tumour cell nuclei following androgen deprivation therapy [61].

Spatial techniques can be used to relate molecular scores to the tumour microenvironment [47]. For example, a TP53 activity score was associated with poor prognosis and imaging mass cytometry was used to demonstrate that a low score was associated with an immunosuppressive tumour microenvironment in bladder cancer [62]. Similar scores could be developed for field cancerisation to inform risk stratification.

## Limitations and Challenges

The cost of spatial transcriptomics investigations remains prohibitive. As a result, spatial studies often select small areas of tissue that are of key interest, such as the tumour edge [63], rather than performing whole organ mapping [13]. For the same reason, these studies are often small in size and exploratory in nature and findings need to be validated in larger studies. This limits translation of findings into the clinical setting. Although sequencing costs are reducing, the cost of spatial techniques remains too high for widespread use in clinical settings [64].

Another challenge is how to identify field cancerised tissue to inform where sample should be taken from. This decision could be informed by histological features, such as early dysplastic changes, by using a molecular marker, such as a gene, or other phenotypic changes. These could be evaluated on a larger scale across the whole organ, as a more cost‐effective interim step to help decide which are the key areas to sample. However, this would introduce a sampling bias and could exclude field cancerised tissues that do not display those changes.

The third challenge is determining what is clinically meaningful field cancerisation. Currently, it is unclear how a cancerised field transforms into cancer, and as a result we do not have a full understanding of the highest risk features of a cancerised field. For example, the total area of a cancerised field may be an important factor as a greater number of cancerised cells could confer a higher chance of transformation into cancer. Alternatively the level of genomic instability could influence the likelihood that a single cell transforms. There may be combinations of driver genes that are required before final transformation can occur, or a specific cellular microenvironment [65]. Once markers of clinically meaningful field cancerisation have been defined, a tool based on the most important markers could be developed to risk stratify patients. This could inform personalised treatment options, such as surveillance frequency or earlier radical treatment, or adjuvant therapies such as immuno‐, radio‐ or chemotherapy for high‐risk patients.

## Conclusions

Spatial omics research on field cancerisation has identified interesting early findings that have informed our understanding of how benign tissues transform into cancerised fields, but the exact mechanisms underlying this as well as the spread of clonal populations remains unclear. Our understanding of spatial organisation of clonal lineages and microenvironments within cancerised fields is growing, and there is an opportunity for spatial profiling of field cancerisation to inform risk stratification and personalised treatment options if the challenges associated with spatial technologies can be overcome.

## Funding

None.

## Disclosure of Interests

None declared.

## Author Contributions

M.H.V.B. and T.A. were responsible for writing the first draft. M.H.V.B. was responsible for reviewing bladder cancer articles and T.A. was responsible for reviewing prostate cancer articles. L.B. and D.J.W. were responsible for supervision. All authors were responsible for revisions.

## Supporting information


**Appendix S1.** Search criteria.
