# Supplementary material for: What spatial omics is teaching us about field cancerisation in prostate and bladder cancer
Source: BJU Int. 2025 Jun 25;136(4):578–89. doi: 10.1111/bju.16830 (PMC12415322; doi:10.1111/bju.16830)
Supplement: Supplementary file 1 — Appendix S1. Search criteria. [file BJU-136-578-s001.docx]

**APPENDIX A: Search criteria**

(Field cancerisation OR field cancerization OR field change OR field effect OR field defect OR normal OR dysplasia OR metaplasia OR hyperplasia OR carcinoma in situ OR Precancerous Conditions [MESH] OR Metaplasia [MESH] OR Hyperplasia [MESH] OR Carcinoma in Situ [MESH] OR Prostatic Intraepithelial Neoplasia [MESH])

AND

(epigenetic* OR genetic* OR proteomic* OR transcriptomic* OR epigenome* OR genome* OR proteome* OR transcriptome* OR methylation OR histone modification OR non-coding RNA OR mutation OR gene expression OR Epigenomics [MESH] OR Genomics [MESH] OR Proteomics [MESH] OR Gene Expression Profiling [MESH] OR Methylation [MESH] OR Histones [MESH] OR Mutation [MESH] OR RNA, Untranslated [MESH])

AND

(spatial OR tissue architecture OR tumor heterogeneity OR regional variation OR visium OR slide-seq OR zenium OR geomx OR merscope OR cosmx OR phenocycler OR cell dive OR mass cytometry imaging OR imaging mass cytometry OR digital spatial profiler OR multiplexed ion beam imaging OR spatial molecular imager OR whole organ mapping)

AND

(Bladder cancer OR Urinary Bladder Neoplasms [MESH] OR Prostatic Neoplasms [MESH])
